# Cochrane corner: improving vaccination coverage among adolescents

**DOI:** 10.11604/pamj.2020.37.160.24127

**Published:** 2020-10-15

**Authors:** Veranyuy Dzeaye Ngah, Kathleen Bime-Fomonyuy Wiysonge, Charles Shey Wiysonge

**Affiliations:** 1 Department of Global Health, Faculty of Medicine and Health Sciences, Stellenbosch University, Cape Town, South Africa,; 2 Cochrane South Africa, South African Medical Research Council, Cape Town, South Africa,; 3 School of Nursing, Faculty of Community and Health Sciences, University of the Western Cape, Cape Town, South Africa,; 4 School of Public Health and Family Medicine, University of Cape Town, Cape Town, South Africa

**Keywords:** Vaccination, adolescents, knowledge

## Abstract

Adolescents refer to individuals in the age group 10 to 19 years. Vaccination of people in this age group offers an opportunity to catch-up on vaccinations missed during childhood, boost waning immunity from childhood vaccines, and provide primary immunity with new vaccines. Vaccination coverage among adolescents is suboptimal worldwide, especially in African countries, and it is unclear which interventions could improve the situation. We focus this commentary on a recent Cochrane review that assessed the effects of interventions to increase vaccination coverage among adolescents. The authors conducted a comprehensive search in multiple peer-reviewed and grey literature databases and identified 16 eligible studies, mostly conducted in high-income countries. The most effective interventions for improving adolescent vaccination coverage included: education of adolescents and their parents about the importance of vaccinations; mandatory vaccination, whereby government enacts laws requiring adolescents to be vaccinated as a pre-condition for school enrolment; and providing a complementary package of educational interventions to adolescents and their parents and healthcare workers. Implementing the evidence from this review would improve adolescent vaccination coverage in Africa. However, given that only one eligible study was conducted in an African country, there is need for African researchers to invest on research in this topic.

## Commentary

Adolescents are people between the ages of 10 and 19 years [1]. Vaccination of adolescents prevents outbreaks of vaccine-preventable diseases. It serves as an opportunity for catch up on vaccine doses missed during childhood and provides primary immunity for new vaccines [2]. Adolescent vaccination coverage is suboptimal in Africa and other low-and-middle-income countries. For example, in 2014, the human papillomavirus (HPV) vaccination coverage was only 1.2% in Africa, compared to 35.6% in North America and 35.9% in Oceania [3]. Some challenges faced by adolescent immunisation programmes in Africa include inadequate knowledge on vaccination among adolescents and their parents and teachers; limited infrastructural and financial resources; and negative attitudes towards adolescent vaccination among adolescents and their parents, teachers, and healthcare workers [4-6]. It is therefore important to evaluate interventions that work to improve uptake of vaccines among adolescents. That was the objective of the Cochrane review by Abdullahi and colleagues, published in January 2020 [2].

Studies eligible for inclusion in the review included randomised trials, non-randomised trials, controlled before-after studies, and interrupted time series studies of adolescents or their parents, teachers, or healthcare providers [2]. The authors searched for publications indexed by May 2019 in multiple electronic databases including, but not limited to, the Cochrane Central Register of Controlled Trials (CENTRAL), MEDLINE, Embase, Scopus, Africa-Wide Information, and Web of Science. In addition, they searched multiple electronic grey literature resources and prospective clinical trial registries. The authors performed duplicate screening, study selection, data extraction, and risk of bias assessment; resolving differences through discussion, consensus, and arbitration. The data were analysed using the Cochrane Review Manager (RevMan) software and the certainty of the evidence was assessed using Grading of Recommendations Assessment, Development and Evaluation (GRADE) [2]. The GRADE approach results in an assessment of the certainty of evidence as high, moderate, low, or very low certainty [7]. High certainty evidence implies that the research provides a very good indication of the likely effect of the intervention, and the likelihood that the true effect will be substantially different is low. Moderate certainty evidence provides a good indication of the likely effect, and the likelihood that the true effect will be substantially different is moderate. Low certainty evidence provides some indication of the likely effect, but the likelihood that the true effect will be substantially different is high. When evidence is of very low certainty, we are uncertain of what the true effect of the intervention could be [7]. In order to express the level of uncertainty in research findings, we often use words such as “probably” and “may” to describe evidence of moderate and low certainty respectively [2].

The comprehensive search identified 29,103 records from which 16 studies were included in the review; 12 randomised trials, three non-randomised trials, and one controlled before-after study. The participants were adolescent girls and boys (in seven studies), parents (in three studies), and healthcare workers (in two studies). Participants in the remaining four studies were a mixture of adolescents, parents, and healthcare providers. Fifteen studies were conducted in high-income countries and only one study was conducted in an African country. Adolescent and parental education on the importance of vaccination led to substantial improvement in adolescent vaccination coverage: risk ratio (RR) 1.43, 95% confidence interval (CI) 1.16 to 1.76; three trials with 1054 participants. Other interventions directed at adolescents and their communities that improved coverage were provision of cash incentives to adolescents (RR 1.45, 95% CI 1.05 to 1.99; one trial with 500 participants) and mandatory vaccination laws requiring adolescents to be vaccinated as a pre-condition for school enrolment (RR 2.94, 95% CI 2.66 to 3.25; one non-randomised trial with 2642 participants). Education of healthcare workers about vaccination, followed by feedback on their performance, increased vaccination by 5.7 percentage points among adolescents. In addition, the review showed that a class-based vaccine delivery strategy increases adolescent vaccination coverage more than an age-based delivery strategy (RR 1.09, 95% CI 1.06 to 1.13; one randomised trial with 5537 participants). The review also found that using a multicomponent intervention directed at adolescents and their parents and healthcare provider’s increases adolescent vaccination coverage (RR 1.41, 95% CI 1.25 to 1.59; one non-randomised trial with 25,869 participants). The intervention consisted of the distribution of short written information on adolescent vaccination to healthcare workers, online training of healthcare workers on adolescent vaccination, educational messages for adolescents and their parents through radio, and simplified information pamphlets on adolescent vaccination for adolescents and their parents. In Table 1 we provide a summary of the evidence from this review, on the effectiveness of the interventions.

**Table 1:** summary of key findings

Recipient-oriented interventions i.e. strategies targeting adolescents and/or their parents and communities						
	**Intervention**	**Comparison**	**Vaccine**	**Sample size**	**Risk ratio (95% CI)**	**GRADE**
1	Health education	Usual practice	HPV	1,054	1.43 (1.16 to 1.76)	High
2	Financial incentive	Usual practice	HPV	500	1.45 (1.05 to 1.99)	Low
3	Mandatory vaccination	Usual practice	Hepatitis B	6,462	3.92 (3.65 to 4.20)	Moderate
Provider-oriented interventions i.e. strategies targeting healthcare workers who provide vaccination services						
	**Intervention**	**Comparison**	**Vaccine**	**Sample size**	**Risk ratio (95% CI)**	**GRADE**
4	Education and performance feedback	Usual practice	HPV	200,000	5.6 percentage* point increase	Low
5	Education, performance feedback, and incentives	Usual practice	HPV	5,786	1.6 ** to 2.2)	Moderate
Health system interventions i.e. delivery, financial, or governance interventions aimed at improving the quality of vaccination services						
6	**Intervention**	**Comparison**	**Vaccine**	**Sample size**	**Risk ratio (95% CI)**	**GRADE**
	Class-based delivery	Age-based delivery	HPV	5537	1.09 (1.06 to 1.13)	Moderate
Multi-component provider and recipient interventions i.e. approaches that include more than one strategy and may target users and/or providers of adolescent vaccination services						
	**Intervention**	**Comparison**	**Vaccine**	**Sample size**	**Risk ratio (95% CI)**	**GRADE**
7	Multi-component provider and recipient intervention	Usual practice	HPV	25,869	1.41 (1.25 to 1.59	Low

CI: confidence interval; HPV: human papillomavirus; GRADE: grading of recommendations assessment, development and evaluation assessment. * The study did not report enough data to calculate the risk ratio, but the intervention increased adolescent vaccination coverage by 5.7 percentage during preventive visits and 5.6 percentage points during acute care visits. ** The effect measure in this study was reported as the odds ratio.

The most effective interventions for improving adolescent vaccination were education and mandatory vaccination. The certainty of this evidence was moderate, implying that, when these interventions are implemented in African countries, their impact needs to be rigorously monitored. The monitoring should ensure that any unfavourable effects on equity during the implementation of these interventions should be redressed quickly and appropriately. Existing evidence shows that adolescents and their parents in Africa have limited knowledge on vaccination but are receptive to knowledge on vaccination when provided [4,8]. As such, the educational strategies in this review would help to improve adolescent vaccination coverage in Africa. Although this review shows mandatory vaccination to have an important effect on vaccination coverage, the intervention may not have the same effect in all settings. The impact would be different, for example, in a context where the reason for low coverage is vaccine hesitancy from a context where the reason for low coverage is access related. Reminders targeting adolescents and/or their parents were not assessed in this review, because they are the focus of another Cochrane review, authored by Jacobson Vann and colleagues [9]. Reminders address barriers such as forgetting or missing appointments and/or not knowing the vaccination schedule. Jacobson Vann and colleagues provide high certainty evidence that reminding adolescents and/or their parents, of upcoming or missed vaccination visits, improves adolescent vaccination coverage (RR 1.29, 95% CI 1.17 to 1.42; 10 randomised trials with 30,868 participants). Telephone calls, text messages, and home visits can be used to deliver reminders in most African settings. Most of the evidence provided above was obtained from high-income countries. These countries differ from African countries not only in terms of financial adequacy but also in terms of educational systems and cultures. In implementing these strategies in African countries, these differences need to be considered by decision makers. Research identifying barriers to adolescent vaccination in Africa is an important first step. This can then be followed by pilot studies implementing the interventions assessed in this review, to determine their feasibility and acceptability in Africa.

## Conclusion

This review showed that educating parents and their adolescent children on vaccination, implementing mandatory vaccinations as a school entry requirement, and using a combination of educational interventions for caregivers and healthcare workers improve adolescent vaccination coverage. Uptake of the evidence from this review in policy and practice in African countries should be accompanied by a robust monitoring and evaluation framework, as most of the evidence was obtained from high-income countries.
